# Underactuated USV path following mechanism based on the cascade method

**DOI:** 10.1038/s41598-022-05456-9

**Published:** 2022-01-27

**Authors:** Mingzhen Lin, Zhiqiang Zhang, Yandong Pang, Hongsheng Lin, Qing Ji

**Affiliations:** 1 College of Weapons Engineering, Naval University of Engineering, Wuhan, 430000 China; 2 College of Political Theories, Naval University of Engineering, Wuhan, 430000 China

**Keywords:** Electronics, photonics and device physics, Electrical and electronic engineering, Mechanical engineering

## Abstract

The path following control under disturbance was studied for an underactuated unmanned surface vehicle (USV) subject to the rudder angle and velocity constraints. For this reason, a variable look-ahead integral line-of-sight (LOS) guidance law was designed on the basis of the disturbance estimation and compensation, and a cascade path following control system was created following the heading control law based on the model prediction. Firstly, the guidance law was designed using the USV three-degree-of-freedom (DOF) motion model and the LOS method, while the tracking error state was introduced to design the real-time estimation of disturbance observer and compensate for the influence of ocean current. Moreover, the stability of the system was analyzed. Secondly, sufficient attention was paid to the rudder angle and velocity constraints and the influence of system delay and other factors in the process of path following when the heading control law was designed with the USV motion response model and the model predictive control (MPC). The moving horizon optimization strategy was adopted to achieve better dynamic performance, effectively overcome the influence of model and environmental uncertainties, and further prove the stability of the control law. Thirdly, a simulation experiment was carried out to verify the effectiveness and advancement of the proposed algorithm. Fourthly, the “Sturgeon 03” USV was used in the lake test of the proposed control algorithm to prove its feasibility in the engineering practices.

## Introduction

Unmanned surface vehicle (USV) has become a hot research topic in the unmanned equipment field at home and abroad because of its intelligent, fast, flexible, stealthy, and weatherproof characteristics. It has enjoyed a very bright future in ocean monitoring, reconnaissance, modern military battle, and security defense in territorial waters^1–3^. USV path following means that a USV under the effect of the control system departs from any initial position, enters the expected path and sails along the expected path with the minimum tracking error^4^. Most USVs have no direct transverse control mechanism, but relies only on the longitudinal propulsive force and steering torque to control the motion, so that they are typical underactuated systems^5^. Due to the lack of control mechanism and the complex and unpredictable marine environment, it is very difficult to control a USV^6^. Therefore, the accurate and stable path following of an underactuated USV in the marine environment becomes a hot topic in the research of USV control.

As a classical and effective guidance algorithm, the line-of-slight (LOS) is not restricted by model, but determines the expected heading of a ship based on its real-time position and planned path. It has been widely applied in the control field since it is slightly affected by high-frequency noise, highly reliable and real time. Reference^7^ designed a cascade control system based on the LOS and feedback linearization method while ignoring the disturbance from the external environment. The system realized the effective path following and was proved to achieve the uniform global K-exponential stability. Based on References^7,8^ further proved the uniform semi-global exponential stability of the system. When the ocean current was known, Reference^9^ put forward an integral LOS guidance law, and introduced the additional longitudinal error to offset the disturbance of ocean current. In this way, the path following control was realized under the disturbance of ocean current. With the designed guidance law, the system was proved to achieve the uniform global asymptotic stability. Reference^10^ further explored the stability of the guidance system and proved the uniform global K-exponential stability of the system. To further enhance the convergence speed of path following while ensuring the stability of the system, Reference^11^ put forth a variable look-ahead integral LOS guidance law, and proved its uniform global K-exponential stability at the equilibrium point. Reference^12^ further verified the uniform semi-global exponential stability of the control system. Do designed a disturbance observer in the Serret-Frenet coordinate system for the effective path following to realize the real-time estimation of and compensation for the disturbance of ocean current. Moreover, the uniform global asymptotic stability of the system was proved^13^. Assuming that the disturbance of ocean current was bounded and varied slowly, Fossen et al. relied on the Lyapunov stability theory and the direct feedback of error to compensate for the drift angle caused by the disturbance including ocean current in a real-time manner. Furthermore, they proved the uniform semi-global exponential stability of the entire system to further improve the real-time and accurate USV path following capability^14^. In Reference^15^, the influence of the complex marine environment was fully considered while designing a path following control system based on the cascade of the variable look-ahead integral LOS guidance algorithm and the adaptive sideslip compensation heading control algorithm. It proved the uniform global asymptotic stability of the system, and presented an offshore following test with ships to verify the effectiveness of algorithms. Considering the influence of USV motion performance, Reference^16^ linearized and discretized the USV three degree of freedom model, considered the control quantity constraints and output constraints, and the path following problem was transformed into a rolling optimization problem under multiple constraints. Reference^17^ further considered the optimization in the quasi infinite time domain based on the predictive control method. However, there is a lack of analysis of the stability of the designed controller, and the linearization of the strong nonlinear model also leads to the decline of model accuracy.

Some achievements have been made at home and abroad with regard to the path following of underactuated USVs, but most of them focus only on the realization of functions and rarely address the constraints on the actuating mechanism in the practical following. Most of controllers are designed on the basis of error feedback control, leading to the delay of control law. In the meantime, most studies pay attention to algorithm design and simulation, but lack the effective verification in the practical scenarios. To address this problem, this paper combines theoretical research, simulation verification, and ship test. At first, the guidance law is designed on the basis of the USV three-DOF motion model and the LOS to analyze the stability of the guidance system. Meanwhile, the longitudinal tracking error is introduced as the state to design the disturbance observer, and realize the real-time estimation and feedback compensation of the disturbance steady-state component. Compared with most output-based feedback compensation algorithms, it is more flexible and controllable. Subsequently, the control law is designed with the USV motion response model and model predictive control (MPC) to address such problems as the instability and large error caused by the rudder angle constraint and system delay in the path following. On this basis, the stability of the control system subject to multiple constraints is further analyzed. After that, a simulation experiment is presented to verify the effectiveness and advancement of the algorithm designed in this paper. At last, a USV ‘Sturgeon’ is used in the lake test for the proposed control algorithm and verify its feasibility in the engineering practices.

The rest of the paper is organized as follows. “Underactuated USV motion model” section establishes a three-degree-of-freedom maneuvering model. The adaptive integral LOS guidance law, model predictive heading control law and disturbance observer are designed in “Path following control system design” section. The problem of path following is defined. And the proof of system stability is given in “Proof of system stability” section. In “Simulation verification and result analysis” section, a simulation experiment is conducted to prove the effectiveness and advancement of the path following system. The feasibility of the system in practical engineering applications is verified by the lake test in “Vessel test and result analysis” section. “Conclusion” section summarizes the work of this paper.

## Underactuated USV motion model

The horizontal three-DOF kinematics and response mathematical model for underactuated USV is built as follows:

1 $$ \dot{x} = u\cos \psi - v\sin \psi $$

2 $$ \dot{y} = u\sin \psi - v\cos \psi $$

3 $$ \psi = \dot{r} $$

4 $$ T\ddot{\psi } + \dot{\psi } = K\delta $$

where ${\varvec{\eta}} = [x,y,\psi ]^{T}$ is the position and heading angle on the horizontal plane in the north-east-down coordinate system; ${{\varvec{\upupsilon}}} = [u,v,r]^{T}$ is the longitudinal and transverse velocities and the heading velocity of the USV in the hull coordinate system; ${\mathbf{J}}(\psi )$ is the conversion matrix from the hull coordinate system to the north-east-down coordinate system; $\delta$ is the input of rudder angle, $T$ is the turning lag index, and $K$ is the turning ability index.

## Path following control system design

### Guidance law design

**Figure 1:**
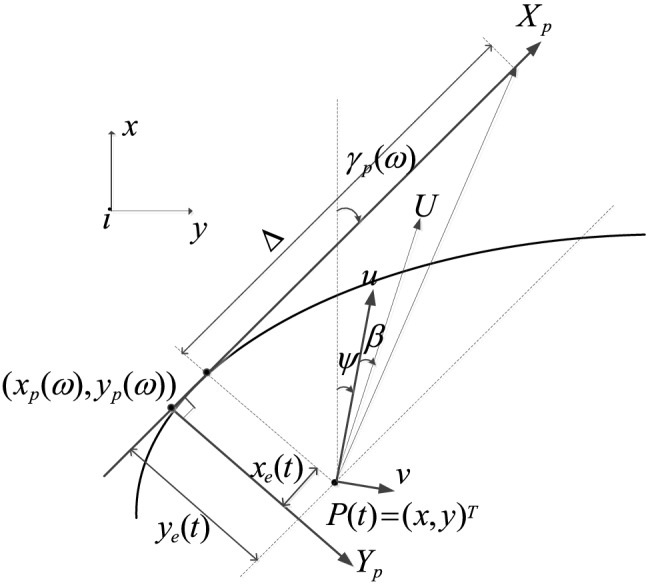
The diagram of LOS guidance.

Based on the integral LOS strategy, the expected heading is expressed as:

5 $$ \psi_{d} = \gamma_{p} (\omega ) - \tan^{ - 1} \left( {\frac{1}{\Delta }\left( {y_{e} (t) + y_{{\text{int}}} } \right)} \right) $$

6 $$ \Delta = (\Delta_{\max } - \Delta_{\min } )e^{{ - \kappa |y_{e} (t)|}} + \Delta_{\min } $$

7 $$ \dot{y}_{e} (t) = U\sin (\psi - \gamma_{p} (\omega ) + \beta ){\kern 1pt} $$

where $\kappa$ is a constant strictly greater than zero; $\Delta_{\max }$ and $\Delta_{\min }$ are the maximum and minimum of the look-ahead distance respectively; $\kappa > 0$; $y_{e}$ is longitudinal tracking error; $\omega$ path update rate, $\beta$ is sideslip angle. When the path following error $y_{e}$ is large, $\mathop {\lim }\nolimits_{{y_{e} (t) \to \infty }} e^{{ - \kappa |y_{e} (t)|}} \approx 0$ and the look-ahead distance is short, so that the USV quickly approaches the given path. When the path following error $y_{e}$ is small, $\mathop {\lim }\nolimits_{{y_{e} (t) \to 0}} e^{{ - \kappa |y_{e} (t)|}} \approx 1$, and the look-ahead distance is long, so that the USV can maintain the stable following on the given path (Fig. 1).

### Control law design

The USV has a limited range of rudder angle. While tracking the heading, the response time of rudder effect may cause the system instability and tracking delay. To address the constraint arising from the range of rudder angle and the influence of rudder effect response in the heading tracking control, the heading controller is designed with predictive control^18,19^.

Equation (4) is transformed into the design model of USV heading controller as follows:

8 $$ \left\{ {\begin{array}{*{20}l} {\dot{\psi } = r} \hfill \\ {\dot{r} = - \frac{1}{T}r + \frac{K}{T}\delta } \hfill \\ \end{array} } \right. $$

where the range of USV rudder angle is $\delta \in [\delta_{\min } ,\delta_{\max } ]$.

The kinematics model in Eq. (8) is written into the state equation as follows:

9 $$ \left\{ {\begin{array}{*{20}l} {\dot{\varvec{x}}_{{\varvec{p}}} (t) = f({\varvec{x}}_{{\varvec{p}}} (t),\delta (t))} \hfill \\ {{\varvec{y}}_{{\varvec{p}}} (t) = {\varvec{Cx}}_{{\varvec{p}}} (t)} \hfill \\ \end{array} } \right. $$

where ${\varvec{x}}_{{\varvec{p}}} (t) = [\psi ,r]^{T}$ is the state variable; $\delta (t)$ is the control input variable; *y*_*p*_(*t*) is the output; and ${\varvec{C}} = {\varvec{I}}_{2 \times 2}$.

Equation (9) is written in the differential form:

10 $$ \left\{ {\begin{array}{*{20}l} {\tilde{\varvec{x}}_{{k{ + }1}} = \varvec{A\tilde{x}}_{k} + \varvec{B}\tilde{\varvec{\delta }}_{k} } \hfill \\ {\tilde{\varvec{y}}_{k} = \varvec{C\tilde{x}}_{k} } \hfill \\ \end{array} } \right. $$

where $${\varvec{A}} = \left[ {\begin{array}{*{20}c} 0 & 1 \\ 0 & { - 1/T} \\ \end{array} } \right]$$ and $${\varvec{B}} = \left[ {\begin{array}{*{20}c} 0 \\ {K/T} \\ \end{array} } \right]$$.

Considering the tracking error and rudder angle constraints, the design objective function is expressed as:

11 $$ J({\varvec{\sigma}},\tilde{\varvec{x}}) = \tilde{\varvec{x}}_{N}^{^{\prime}} \varvec{P\tilde{x}}_{N} + \sum\limits_{k = 0}^{N - 1} {\tilde{\varvec{x}}_{k}^{\prime } } \varvec{Q\tilde{x}}_{k} + {\varvec{\delta}}_{k}^{\prime } \varvec{R}\varvec{\delta }_{k} $$

12 $$ {\varvec{\delta}}_{\min } \le {\varvec{\delta}}_{k} \le {\varvec{\delta}}_{\max } {\kern 1pt} ,k = 0, \ldots ,N - 1 $$

where *N* is the moving horizon optimization predicted length; $P$, $Q$ and $R$ are the weight coefficient matrices and their diagonal elements are all greater than 0. In the objective function, the first term is the terminal constraint penalty; the second term reflects the capability of tracking the expected heading; and the third term reflects the demand for the stable variation of rudder angle.

The optimization problem defined in Eqs. (11) and (12) is converted into the quadratic programming problem and then solved.

Equation (10) is rewritten into

13 $$ \tilde{\varvec{x}}_{k} { = }{\varvec{A}}^{k} \tilde{\varvec{x}}_{0} + \sum\limits_{j = 0}^{k - 1} {{\varvec{A}}^{j} } \varvec{B}\varvec{\delta }_{k - 1 - j} . $$

Equation (11) is rewritten into

14 $$ J({\varvec{\sigma}},\tilde{\varvec{x}}) = \tilde{\varvec{x}}^{\prime } \varvec{Q\tilde{x}} + {\varvec{\chi}}^{\prime } \overline{\varvec{Q}}{\varvec{\chi}} + {\varvec{\sigma}}^{\prime } \overline{\varvec{R}}\varvec{\sigma } $$

where $${\varvec{\chi}} = \left[ {\begin{array}{*{20}c} {\tilde{\varvec{x}}_{0} } & {\tilde{\varvec{x}}_{1} } & { \cdot \cdot \cdot } & {\tilde{\varvec{x}}_{N - 1} } \\ \end{array} } \right]^{T}$$, $$\overline{\varvec{Q}} = diag[\underbrace {{{\varvec{Q}},{\varvec{Q}}, \cdots {\varvec{Q}}}}_{N - 1},{\varvec{P}}]$$, $$\overline{\varvec{R}} = diag[\underbrace {{{\varvec{R}},{\varvec{R}}, \cdots {\varvec{R}}}}_{N}]$$, $${\varvec{\chi}} = \varvec{\phi \sigma }{ + }\varvec{{E}\tilde{x}}$$, $${\varvec{\phi}} = \left[ {\begin{array}{*{20}c} {{\varvec{B}}_{d} } & 0 & \cdots & 0 \\ {{\varvec{A}}_{d} {\varvec{B}}_{d} } & {{\varvec{B}}_{d} } & \cdots & 0 \\ \vdots & \vdots & \ddots & \vdots \\ {{\varvec{A}}_{d}^{N - 1} {\varvec{B}}_{d} } & {{\varvec{A}}_{d}^{N - 2} {\varvec{B}}_{d} } & \cdots & {{\varvec{B}}_{d} } \\ \end{array} } \right]$$.

Equation (14) is further converted to obtain

15 $$ \begin{aligned} J({\varvec{\sigma}},\tilde{\varvec{x}}) & = (\varvec{\phi \sigma } + \varvec{{E}\tilde{x}})^{\prime } \overline{\varvec{Q}}(\varvec{\phi \sigma } + {\varvec{E}\tilde{\varvec{x}}}){\kern 1pt} + {\varvec{\sigma}}^{\prime } {\overline{\varvec{R}}\varvec{\sigma }} + \tilde{\varvec{x}}^{\prime } {\varvec{Q}\tilde{\varvec{x}}} \\ & = {\varvec{\sigma}}^{\prime } (\overline{\varvec{R}} + {\varvec{\phi}}^{\prime } {\overline{\varvec{Q}}\varvec{\phi }}){\varvec{\sigma}} + \tilde{\varvec{x}}^{\prime } \varvec{E}^{\prime } {\overline{\varvec{Q}}\varvec{\phi \sigma }} + \tilde{\varvec{x}}^{\prime } ({\varvec{Q}} + \varvec{E}^{\prime } \overline{\varvec{Q}}{\varvec{E}})\tilde{\varvec{x}} \\ & = \frac{1}{2}{\varvec{\sigma}}^{\prime } \varvec{\varPhi \sigma } + \tilde{\varvec{x}}^{\prime } \varvec{\varPsi \sigma } + \frac{1}{2}\tilde{\varvec{x}}^{\prime } {\varvec{\varUpsilon }\tilde{\varvec{x}}} \\ \end{aligned} $$

where $${\varvec{\varPhi}}= 2(\overline{\varvec{R}} + {\varvec{\phi}}^{\prime } {\overline{\varvec{Q}}\varvec{\phi }})$$, $${\varvec{\varPsi}}= {2}\varvec{E}^{\prime } {\overline{\varvec{Q}}{\varvec{E}}}$$ and $${\varvec{\varUpsilon}}= ({\varvec{Q}} + \varvec{E}^{\prime } {\overline{\varvec{Q}}{\varvec{E}}})$$.

Equation (12) for the constraints is written as

16 $$ \varvec{G\sigma } \le {\varvec{\tau}} $$

where $${\varvec{G}} = [{\varvec{I}}_{{\varvec{N}}} - {\varvec{I}}_{{\varvec{N}}} ]^{T}$$ and $${\varvec{\tau}} = [\underbrace {{{\varvec{\delta}}_{\max } \cdots {\varvec{\delta}}_{\max } }}_{N}\;{\kern 1pt} \underbrace {{ - {\varvec{\delta}}_{\min } \cdots - {\varvec{\delta}}_{\min } }}_{N}]$$.

To sum up, the optimization problem is converted into the quadratic programming problem subject to multiple constraints as follows:

17 $$ \left\{ {\begin{array}{*{20}l} {\mathop {\min }\limits_{{\varvec{\sigma}}} \frac{1}{2}{\varvec{\sigma}}^{\prime } \varvec{\varPhi \sigma + \tilde{x}}^{\prime } \varvec{\varPsi \sigma }} \hfill \\ {\varvec{G\sigma } \le {\varvec{\tau}}} \hfill \\ \end{array} } \right. $$

Since ${\varvec{\varPhi}}\ge 0$, the quadratic programming has a solution for each non-negative weight matrix $P$, $Q$ or $R$.

### Disturbance observer design

Equations (5) and (7) are used to obtain

18 $$ \begin{aligned} \dot{y}_{e} & = U\sin (\tilde{\psi } + \psi_{d} - \gamma_{{\text{p}}} + \beta ) = U\sin (\tilde{\psi } + \tan^{ - 1} \left( { - \frac{1}{\Delta }({\text{y}}_{{\text{e}}} + {\text{y}}_{{\text{int}}} )} \right) + \beta ) \\ & = U\sin \left( {\tilde{\psi } + \tan^{ - 1} \left( { - \frac{1}{\Delta }({\text{y}}_{{\text{e}}} + {\text{y}}_{{\text{int}}} )} \right)} \right)\cos \beta \\ & \quad + \;U\cos \left( {\tilde{\psi } + \tan^{ - 1} \left( { - \frac{1}{\Delta }({\text{y}}_{{\text{e}}} + {\text{y}}_{{\text{int}}} )} \right)} \right)\sin \beta . \\ \end{aligned} $$

The sideslip angle caused by the disturbance of ocean current is very small, i.e. $\sin \beta \approx \beta$, $\cos \beta \approx 1$.

With Eq. (18), it is further derived that

19 $$ \begin{aligned} \dot{y}_{e} & = U\sin \left( {\tilde{\psi } + \tan^{ - 1} \left( { - \frac{1}{\Delta }({\text{y}}_{{\text{e}}} + {\text{y}}_{{\text{int}}} )} \right)} \right){\kern 1pt} {\kern 1pt} \\ & \quad + \;U\cos \left( {\tilde{\psi } + \tan^{ - 1} \left( { - \frac{1}{\Delta }({\text{y}}_{{\text{e}}} + {\text{y}}_{{\text{int}}} )} \right)} \right)\beta \\ & = U\sin (\tilde{\psi })\cos \left( {\tan^{ - 1} \left( { - \frac{1}{\Delta }({\text{y}}_{{\text{e}}} + {\text{y}}_{{\text{int}}} )} \right)} \right) \\ & \quad + \;U\cos (\tilde{\psi })\sin \left( {\tan^{ - 1} \left( { - \frac{1}{\Delta }({\text{y}}_{{\text{e}}} + {\text{y}}_{{\text{int}}} )} \right)} \right){\kern 1pt} \\ & \quad + \;\beta U\cos (\tilde{\psi })\cos \left( {\tan^{ - 1} \left( { - \frac{1}{\Delta }({\text{y}}_{{\text{e}}} + {\text{y}}_{{\text{int}}} )} \right)} \right) \\ & \quad - \;\beta U\sin (\tilde{\psi })\sin \left( {\tan^{ - 1} \left( { - \frac{1}{\Delta }({\text{y}}_{{\text{e}}} + {\text{y}}_{{\text{int}}} )} \right)} \right). \\ \end{aligned} $$

Moreover, there is

20 $$ \sin \left( {\arctan \left( { - \frac{1}{\Delta }\left( {y_{e} - y_{{\text{int}}} } \right)} \right)} \right) = - \frac{{y_{e} + y_{{\text{int}}} }}{{\sqrt {\Delta^{2} + (y_{e} (t) + y_{{\text{int}}} )^{2} } }} $$

21 $$ \cos \left( {\arctan \left( { - \frac{1}{\Delta }\left( {y_{e} - y_{{\text{int}}} } \right)} \right)} \right) = - \frac{\Delta }{{\sqrt {\Delta^{2} + (y_{e} (t) + y_{{\text{int}}} )^{2} } }}. $$

Equations (20) and (21) are substituted into Eq. (19) to obtain

22 $$ \begin{aligned} \dot{y}_{e} & = U\sin (\tilde{\psi })\frac{\Delta }{{\sqrt {\Delta^{2} + ({\text{y}}_{{\text{e}}} + {\text{y}}_{{\text{int}}} )^{2} } }}{\kern 1pt} {\kern 1pt} - U\cos (\tilde{\psi })\frac{{{\text{y}}_{{\text{e}}} + {\text{y}}_{{\text{int}}} }}{{\sqrt {\Delta^{2} + ({\text{y}}_{{\text{e}}} + {\text{y}}_{{\text{int}}} )^{2} } }} \\ & \quad + \;\beta U\sin (\tilde{\psi })\frac{{{\text{y}}_{{\text{e}}} + {\text{y}}_{{\text{int}}} }}{{\sqrt {\Delta^{2} + ({\text{y}}_{{\text{e}}} + {\text{y}}_{{\text{int}}} )^{2} } }}{\kern 1pt} + \beta U\cos (\tilde{\psi })\frac{\Delta }{{\sqrt {\Delta^{2} + ({\text{y}}_{{\text{e}}} + {\text{y}}_{{\text{int}}} )^{2} } }} \\ & = - \frac{{({\text{y}}_{{\text{e}}} + {\text{y}}_{{\text{int}}} )}}{{\sqrt {\Delta^{2} + ({\text{y}}_{{\text{e}}} + {\text{y}}_{{\text{int}}} )^{2} } }}U{\kern 1pt} + \frac{\beta \Delta }{{\sqrt {\Delta^{2} + ({\text{y}}_{{\text{e}}} + {\text{y}}_{{\text{int}}} )^{2} } }}U + U\phi (y_{e} ,\tilde{\psi })\tilde{\psi } \\ \end{aligned} $$

where

23 $$ \phi ({\text{y}}_{{\text{e}}} ,\tilde{\psi }) = U\frac{{\sin (\tilde{\psi })}}{{\tilde{\psi }}}\frac{{\Delta + \beta ({\text{y}}_{{\text{e}}} + {\text{y}}_{{\text{int}}} )}}{{\sqrt {\Delta^{2} + ({\text{y}}_{{\text{e}}} + {\text{y}}_{{\text{int}}} )^{2} } }}{\kern 1pt} - U\frac{{\cos (\tilde{\psi }) - 1}}{{\tilde{\psi }}}\frac{{({\text{y}}_{{\text{e}}} + {\text{y}}_{{\text{int}}} ) - \beta \Delta }}{{\sqrt {\Delta^{2} + ({\text{y}}_{{\text{e}}} + {\text{y}}_{{\text{int}}} )^{2} } }}. $$

Since $\left| {\frac{{\sin (\tilde{\psi })}}{{\tilde{\psi }}}} \right| \le 1$, $\left| {\frac{{\cos (\tilde{\psi }) - 1}}{{\tilde{\psi }}}} \right| \le 0.73$ and $\left| {\frac{{\Delta + \beta ({\text{y}}_{{\text{e}}} + {\text{y}}_{{\text{int}}} )}}{{\sqrt {\Delta^{2} + ({\text{y}}_{{\text{e}}} + {\text{y}}_{{\text{int}}} )^{2} } }}} \right| \le \left| {1 + \beta } \right|$, it is found that $\left| {\phi ({\text{y}}_{{\text{e}}} ,\tilde{\psi })} \right| \le 1.73\left| {1 + \beta } \right|$ is bounded.

The disturbance of ocean current is taken into account for its real-time observation and compensation to obtain

24 $$ \Omega = \frac{\beta \Delta }{{\sqrt {\Delta^{2} + ({\text{y}}_{{\text{e}}} + {\text{y}}_{{\text{int}}} )^{2} } }}U. $$

It is known that $\Omega \le \beta U$ is bounded. At the stage of stable following, $\Omega \approx \beta \sqrt {u_{d}^{2} + v_{c}^{2} }$ (where $v_{c}$ is the transverse ocean current component in the S-F coordinate system). The sideslip angle caused by the disturbance of ocean current is very small and varies very slowly, so that it is regarded as a constant within a short period. Therefore, there is $\dot{\beta } \approx 0$, and then $\dot{\Omega } \approx 0$.

Considering the observability of the transverse error ${\text{y}}_{{\text{e}}}$, the adaptive disturbance observer is designed as follows:

25 $$ \left\{ {\begin{array}{*{20}l} {\dot{\hat{y}}_{e} = - \frac{{U(\hat{\text{y}}_{{\text{e}}} + {\text{y}}_{{\text{int}}} )}}{{\sqrt {\Delta^{2} + ({\text{y}}_{{\text{e}}} + {\text{y}}_{{\text{int}}} )^{2} } }} + \hat{\Omega } + K_{1} ({\text{y}}_{{\text{e}}} - \hat{\text{y}}_{{\text{e}}} )} \hfill \\ {\dot{\hat{\Omega }} = K_{2} ({\text{y}}_{{\text{e}}} - \hat{\text{y}}_{{\text{e}}} )} \hfill \\ \end{array} } \right. $$

where $K_{1} = K_{1c} e^{{(\sigma \left| {\tilde{\text{y}}_{{\text{e}}} } \right|)^{ - 1} }}$, $K_{2} = K_{2c} e^{{(\sigma \left| {\tilde{\text{y}}_{{\text{e}}} } \right|)^{ - 1} }}$, $K_{1c}$ and $K_{2c}$ are both the constants greater than zero; $\tilde{y}_{e} = y_{e} - \hat{y}_{e}$ is the state estimation error of the transverse error.

When $y_{e} { = }0$, the compensation integral term is obtained as

26 $$ \hat{\text{y}}_{{\text{int}}} = \Delta \frac{{\hat{\Omega }/{\text{U}}}}{{\sqrt {1 - (\hat{\Omega }/{\text{U}})^{2} } }}. $$

Obviously, the disturbance observation subsystem achieves the uniform global asymptotic stability and uniform semi-global exponential stability at the equilibrium point.

## Proof of system stability

The stability of the guidance subsystem is proved as follows: When the transverse component of the distance is accurately estimated, and $$\zeta = \left[ {\begin{array}{*{20}c} {\tilde{u}} & {\tilde{\psi }} & {\dot{\tilde{\psi }}} \\ \end{array} } \right]^{T} = 0_{3 \times 1}$$, the guidance subsystem $\sum 1$ has the uniform global asymptotic stability and uniform semi-global exponential stability at the equilibrium point $(y_{e} ,\zeta ) = (0,0,0,0)$.

### Proof

Equation (22) is rewritten into

27 $$ \sum {1:} \, \dot{y}_{e} = - \frac{{({\text{y}}_{{\text{e}}} + {\text{y}}_{{\text{int}}} )}}{{\sqrt {\Delta^{2} + ({\text{y}}_{{\text{e}}} + {\text{y}}_{{\text{int}}} )^{2} } }}U{\kern 1pt} + \frac{\beta \Delta }{{\sqrt {\Delta^{2} + ({\text{y}}_{{\text{e}}} + {\text{y}}_{{\text{int}}} )^{2} } }}U + g({\text{t}},{\text{y}}_{{\text{e}}} ,\zeta )\zeta $$

where $$g({\text{t}},{\text{y}}_{{\text{e}}} ,\zeta ) = \left[ {\begin{array}{*{20}c} 0 & {U\phi ({\text{y}}_{{\text{e}}} ,\tilde{\psi })} & 0 \\ \end{array} } \right]$$.

If the disturbance can be accurately estimated, and the stable tracking of heading and velocity can be realized, the system $\sum 1$ can be written as

28 $$ \sum {1^{\prime } :} \, \dot{y}_{e} = - \frac{{{\text{y}}_{{\text{e}}} }}{{\sqrt {\Delta^{2} + ({\text{y}}_{{\text{e}}} + {\text{y}}_{{\text{int}}} )^{2} } }}U. $$

The system $\sum 1$ achieves the uniform global asymptotic stability and uniform semi-global exponential stability at the equilibrium point $(y_{e} (t),\zeta ) = (0,0,0,0)$.

The heading control subsystem subject to the rudder angle constraint is proved as follows:

In the terminal inequality constraint $\tilde{\varvec{x}}(k + N) \in \Omega$, $\Omega$ represents a neighborhood adjacent to the origin and $\Omega$ is a positive invariant region of the controlled system and satisfies $({\varvec{A}} + {\varvec{BL}})\tilde{\varvec{x}}(k) \in \Omega ,\quad \forall \tilde{\varvec{x}} \in \Omega$.

29 $$ \tilde{\varvec{x}}(k + 1) = ({\varvec{A}} + {\varvec{BL}})\tilde{\varvec{x}}(k) $$

### Lemma

Assuming that $P > 0$ is the positive definite solution of the Lyapunov equation ${\varvec{P}} = ({\varvec{A}} + {\varvec{BL}})^{T} P({\varvec{A}} + {\varvec{AL}}) + ({\varvec{C}}^{T} {\varvec{QC}} + {\varvec{L}}^{T} {\varvec{RL}})$, there is $\exists \alpha > 0$, so that the system (29) satisfies the control constraint in $\Omega = \{ \tilde{\varvec{x}}_{k} \in R^{n} |\tilde{\varvec{x}}_{k}^{T} \varvec{P\tilde{x}}_{k} \le \alpha \}$.

The problem is redefined as

30 $$ \left\{ {\begin{array}{*{20}l} {\mathop {\min }\limits_{{\Gamma_{k} }} J({\varvec{\sigma}},\tilde{\varvec{x}})} \hfill \\ {{\varvec{\tau}}_{\min } \le {\varvec{\tau}}_{k} \le {\varvec{\tau}}_{\max } {\kern 1pt} ,k = 0, \ldots ,N - 1} \hfill \\ {\tilde{\varvec{x}}(k + N) \in \Omega } \hfill \\ \end{array} } \right.. $$

### Theorem

If *k* = 0, the optimization problem (30) has solutions. Among them, $P > 0$ is the solution of the Lyapunov equation ${\varvec{P}} = ({\varvec{A}} + {\varvec{BL}})^{T} P({\varvec{A}} + {\varvec{BL}}) + ({\varvec{C}}^{T} {\varvec{QC}} + {\varvec{L}}^{T} {\varvec{RL}})$. According to the lemma definition, $\Omega$ satisfies the rudder angle constraint. When external disturbance and model error are ignored, the optimization problem has solutions for $\forall k > 0$, and the system (29) has the uniform global asymptotic stability.

### Proof

The solution of the problem (30) at the time *k* is:

31 $${\varvec{\varGamma}}_{k}^{ * } = \left[ {\begin{array}{*{20}c} {{\varvec{\tau}}^{ * } (k|k)} \\ {{\varvec{\tau}}^{ * } (k + 1|k)} \\ \vdots \\ {{\varvec{\tau}}^{ * } (k + N - 1|k)} \\ \end{array} } \right]. $$

It satisfies the rudder angle constraint. The corresponding predictive state sequence is $\left\{ {\tilde{\varvec{x}}^{ * } (k + 1|k),\tilde{\varvec{x}}^{ * } (k + 2|k), \ldots ,\tilde{\varvec{x}}^{ * } (k + N|k)} \right\}$, and satisfies the terminal constraint equation. The optimized value is:

32 $$ J_{k}^{ * } = \left\| {\tilde{\varvec{x}}^{ * } (k + N|k)} \right\|_{P}^{2} + \sum\limits_{i = 0}^{N - 1} {\left( {\left\| {\tilde{\varvec{y}}^{ * } (k + i)} \right\|_{Q}^{2} + \left\| {{\varvec{\delta}}^{*} (k + i)} \right\|_{R}^{2} } \right)} . $$

The closed-loop control is:

33 $$ {\varvec{\delta}}(k) = {\varvec{\delta}}^{ * } (k|k). $$

It is substituted into the system Eq. (29) to obtain

34 $$ \tilde{\varvec{x}}(k + 1) = {\varvec{A}\tilde{\varvec{x}}}(k) + \varvec{B\delta }^{ * } (k|k) $$

and there is $\tilde{\varvec{x}}(k{ + }1) = \tilde{\varvec{x}}^{ * } (k + 1|k)$.

The rudder angle sequence is selected at the time *k* + 1 as follows:

35 $${\varvec{\varGamma}}_{{k{ + }1}} = \left[ {\begin{array}{*{20}c} {{\varvec{\delta}}(k{ + }1|k{ + }1)} \\ {{\varvec{\delta}}(k + 2|k{ + }1)} \\ \vdots \\ {{\varvec{\delta}}(k + N - 1|k{ + }1)} \\ {{\varvec{\delta}}(k + N|k{ + }1)} \\ \end{array} } \right] = \left[ {\begin{array}{*{20}c} {{\varvec{\delta}}^{ * } (k{ + }1|k)} \\ {{\varvec{\delta}}^{ * } (k + 2|k)} \\ \vdots \\ {{\varvec{\delta}}^{ * } (k + N - 1|k)} \\ {\varvec{L\tilde{x}}^{ * } (k + N|k)} \\ \end{array} } \right]. $$

Since $\tilde{\varvec{x}}^{ * } (k + N|k) \in \Omega$, there is

36 $$ {\varvec{\delta}}_{\min } \le \varvec{L\tilde{x}}^{ * } (k + N|k) \le {\varvec{\delta}}_{\max } . $$

Therefore, it satisfies the rudder angle constraint.

Since

37 $$ \begin{aligned} \tilde{\varvec{x}}(k{ + }1{ + }N|k{ + }1) & = \varvec{A\tilde{x}}(k{ + }N|k{ + }1) + \varvec{B\tau }(k{ + }N|k{ + }1) \\ & = ({\varvec{A}} + {\varvec{BL}})\tilde{\varvec{x}}^{ * } (k{ + }N|k). \\ \end{aligned} $$

The corresponding state sequence is:

38 $$ \tilde{\varvec{x}}(k{ + }1{ + }N|k{ + }1) = \left\{ {\begin{array}{*{20}l} {\tilde{\varvec{x}}^{ * } (k{ + }1{ + }i|k),} \hfill & {i = 0, \ldots ,N - 1} \hfill \\ {({\varvec{A}} + {\varvec{BL}})\tilde{\varvec{x}}^{ * } (k{ + }N|k),} \hfill & {i = N} \hfill \\ \end{array} } \right. $$

and $\tilde{\varvec{x}}^{ * } (k + N|k) \in \Omega$, so that there is

39 $$ ({\varvec{A}} + {\varvec{BL}})\tilde{\varvec{x}}^{ * } (k{ + }N|k) \in \Omega . $$

The selected control sequence satisfies the terminal inequality constraint.

Equation (35) is substituted into Eq. (30) to obtain:

40 $$ \begin{aligned} J_{{k{ + }1}} & = \left\| {\tilde{\varvec{x}}(k{ + }1{ + }N|k{ + }1)} \right\|_{P}^{2} { + }{\kern 1pt} \sum\limits_{i = 0}^{N - 1} {(\left\| {\tilde{\varvec{y}}(k{ + 1 + }i|k{ + }1)} \right\|_{Q}^{2} { + }\left\| {{\varvec{\delta}}(k{ + 1 + }i|k{ + }1)} \right\|_{R}^{2} )} \\ & { = }\left\| {({\varvec{A}} + {\varvec{BL}})\tilde{\varvec{x}}^{ * } (k{ + }N|k)} \right\|_{P}^{2} { + }{\kern 1pt} {\kern 1pt} \sum\limits_{i = 0}^{N - 2} {(\left\| {\tilde{\varvec{y}}^{ * } (k{ + 1 + }i|k)} \right\|_{Q}^{2} { + }\left\| {{\varvec{\delta}}^{ * } (k{ + 1 + }i|k)} \right\|_{R}^{2} )} {\kern 1pt} \\ & \quad + \;\left\| {\varvec{C\tilde{x}}^{ * } (k{ + }N|k)} \right\|_{Q}^{2} { + }\left\| {\varvec{L\tilde{x}}^{ * } (k{ + }N|k)} \right\|_{R}^{2} \\ & = \left\| {\tilde{\varvec{x}}^{ * } (k{ + }N|k)} \right\|_{P}^{2} { + }{\kern 1pt} {\kern 1pt} \sum\limits_{i = 1}^{N - 1} {(\left\| {\tilde{\varvec{y}}^{ * } (k{ + }i|k)} \right\|_{Q}^{2} { + }\left\| {{\varvec{\delta}}^{ * } (k{ + }i|k)} \right\|_{R}^{2} )} \\ & = \left\| {\tilde{\varvec{x}}^{ * } (k{ + }N|k)} \right\|_{P}^{2} { + }{\kern 1pt} \sum\limits_{i = 0}^{N - 1} {(\left\| {\tilde{\varvec{y}}^{ * } (k{ + }i|k)} \right\|_{Q}^{2} { + }\left\| {{\varvec{\delta}}^{ * } (k{ + }i|k)} \right\|_{R}^{2} )} {\kern 1pt} {\kern 1pt} \\ & \quad - \;\left\| {\tilde{\varvec{y}}^{ * } (k|k)} \right\|_{Q}^{2} - \left\| {{\varvec{\delta}}^{ * } (k|k)} \right\|_{R}^{2} . \\ \end{aligned} $$

Let $\tilde{\varvec{y}}^{ * } (k|k) = \varvec{C\tilde{x}}_{k}$, ${\varvec{\delta}}^{ * } (k|k) = {\varvec{\delta}}_{k}$, it is obtained that

41 $$ J_{{k{ + }1}}^{{}} { = }J_{k}^{ * } - \left\| {\varvec{C\tilde{x}}_{k} } \right\|_{Q}^{2} - \left\| {{\varvec{\delta}}_{k} } \right\|_{R}^{2} . $$

Therefore, the objective function is bounded.

The rudder angle control sequence is the feasible solution of the optimization problem. If the optimization problem (30) has an optimized solution, the optimized solution must be superior to the feasible solution. Therefore, it is obtained that:

42 $$ J_{{k{ + }1}}^{ * } \le J_{{k{ + }1}}^{{}} \le J_{k}^{ * } - \left\| {\varvec{C\tilde{x}}_{k} } \right\|_{Q}^{2} . $$

When $\tilde{\varvec{x}} = 0$, ${\varvec{\delta}} = 0$. At this time, ${\varvec{\delta}}^{ * } = 0$ is the feasible solution of the optimization problem, and $J^{*} = 0$. For $\forall k \ge 0$, there is $J_{k}^{ * } \ge 0$, which monotonically decreases, and $J_{k}^{ * }$ is the minimum at $\tilde{\varvec{x}} = 0$.

To prove the continuity of $J_{k}^{ * }$ at $\tilde{\varvec{x}} = 0$, let $\tilde{\varvec{x}} \in \Omega$ at the equilibrium point. The feasible solution is selected as

43 $$ {\varvec{\delta}}_{k} = {\varvec{L}\tilde{\varvec{x}}}_{k} \quad {\varvec{L}} \ge 0. $$

Considering the randomness of $\tilde{\varvec{x}} \in \Omega$, for the given $\varepsilon > 0$, $\ell = \ell (\varepsilon ) > 0$, so that $\forall \left\| {\tilde{\varvec{x}}} \right\| < \ell$, and then $J_{k}^{{}} < \varepsilon$. The optimal solution is certainly superior to the feasible solution, and $J_{k}^{ * } \ge 0$, so that we obtain

44 $$ 0 \le J_{k}^{ * } \le J_{k} < \varepsilon . $$

Therefore, $J_{k}^{ * }$ is continuous at $\tilde{\varvec{x}} = 0$. Based on the Theorem 5.3 in Reference^20^, it is found that $J_{k}^{ * }$ is a Lyapunov function of the closed-loop system (29), so that the closed-loop system can realize the uniform global asymptotic stability at the equilibrium point. As revealed in the stability analysis, the cascade control system has the uniform global asymptotic stability at the equilibrium point.

## Simulation verification and result analysis

In simulation and testing, the main configuration parameters of the industrial computer are as follows: CPU intel core i5-3610, host frequency 2.7 GHz, 8G memory, and 64-bit win7 operating system.

### Heading controller simulation

**Figure 2:**
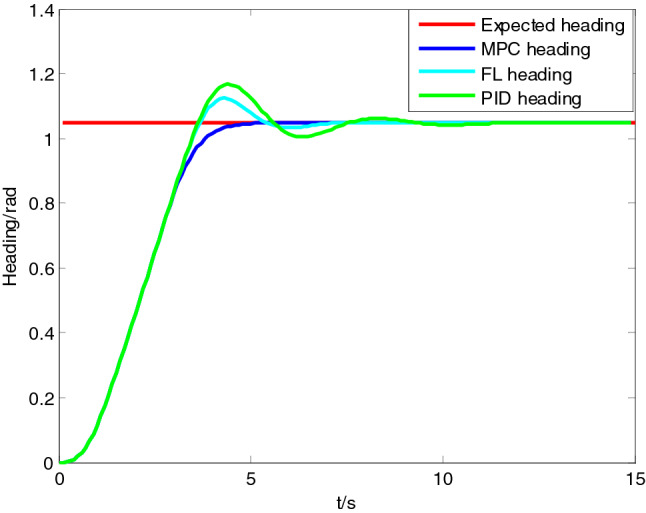
Comparison of course tracking effect.

**Figure 3:**
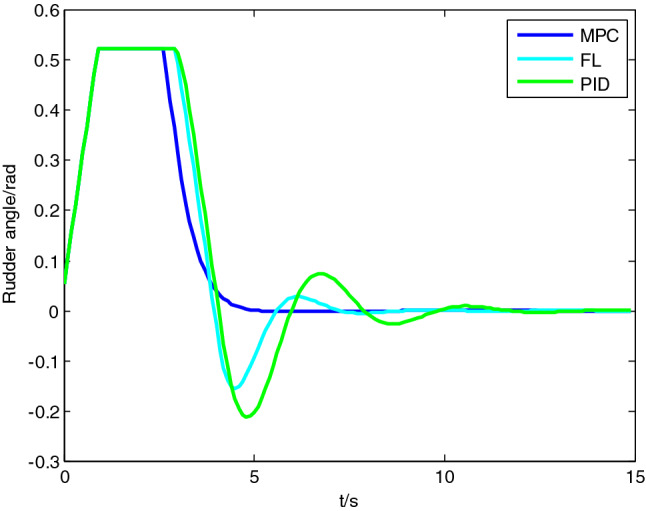
Comparison of rudder angle change curve.

The model parameters of ‘Sturgeon’ USV were $K = 0.738$ and $T = 0.295$. The sampling cycle of system was $T_{s} = 200\;{\text{ms}}$; the prediction cycle of MPC was $40T_{s}$; the control cycle was $2T_{s}$; and the diagonal elements of weight matrixes $Q$ and $R$ were 1 and 10 respectively. According to the feedback linearization (FL) method in Reference^21,22^, heading control law parameters $k_{\psi }$ and $k_{r}$ were 5 and 1 respectively. PID control law had proportional factor $K_{p} = 0.45$, integral factor $K_{i} = 0.001$, and differentiated factor $K_{d} = 0.15$. The expected heading was 30°, and the original heading and rudder angle were both 0. The range of rudder angle was $\left[ { - \frac{\pi }{6}rad,\frac{\pi }{6}rad} \right]$. The heading tracking result and controller quantity are presented in Figs. 2 and 3 respectively.

As revealed in the simulation results, the rise time, settling time, and percent overshoot of the heading controller based on PID are around 3.5 s, 9.5 s and 17% respectively, and those of the heading controller based on FL are around 3.5 s, 7.5 s and 12% respectively, while those of the heading controller based on MPC are around 4 s, 5 s and 0.1% respectively. All three algorithms could realize the stable heading tracking. Figure 2 clearly reveals that, the PID controller and the FL controller generate very high overshoot and oscillation at the stage of convergence (around 4.5 s), and need longer time to become stable. The heading controller based on MPC rarely has overshoot at the same rate of convergence, and is able to realize stable tracking within a short period, so that its control effect is much better. As clearly indicated in Fig. 3 rudder angle curve, the heading controller based on MPC could lower the rudder angle in advance before reaching the expected heading, so as to reduce overshoot, while the heading controllers based on PID and FL have obvious delay, causing the instability of the system. And the optimal solutions given by MPC algorithm do not exceed the set limits of rudder angle in the simulated conditions.

Combining algorithm principles to further analyze the results of simulation and comparison experiments, we can get the following conclusions.

1. The MPC controller itself has a feedforward compensation mechanism with the moving horizon optimization strategy, and could achieve better convergence and lower overshoot. While the PID controller and the FL controller take compensation for existing error as tuning mechanism and may easily lead to much overshoot because of rudder delay and vessel inertia.
2. The MPC controller obtains optimal control sequence based on quadratic programming, therefore, the solution obtained meets the constraint of rudder angle, which is difficult to achieve with the other two controllers.
3. Compared with the PID controller and the FL controller, the MPC controller obtains better tracking performance, but adds computational load in the implementation.

### Path following simulation

**Figure 4:**
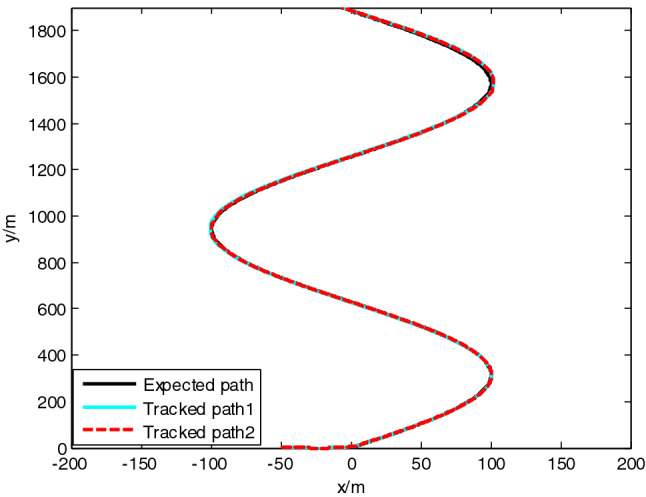
Path following.

**Figure 5:**
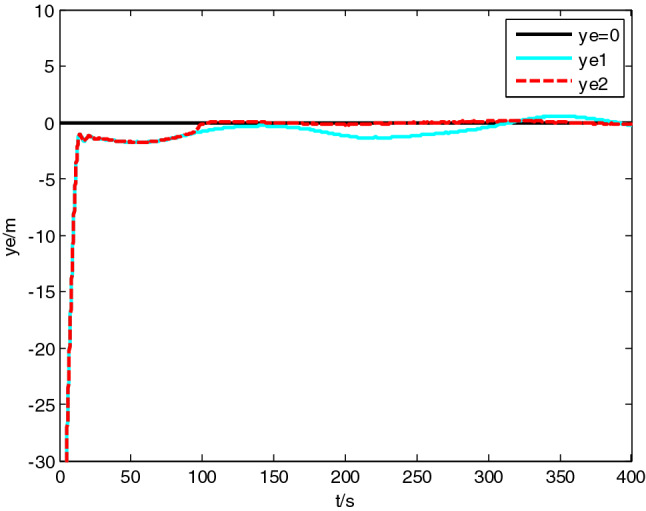
Longitudinal tracking error.

**Figure 6:**
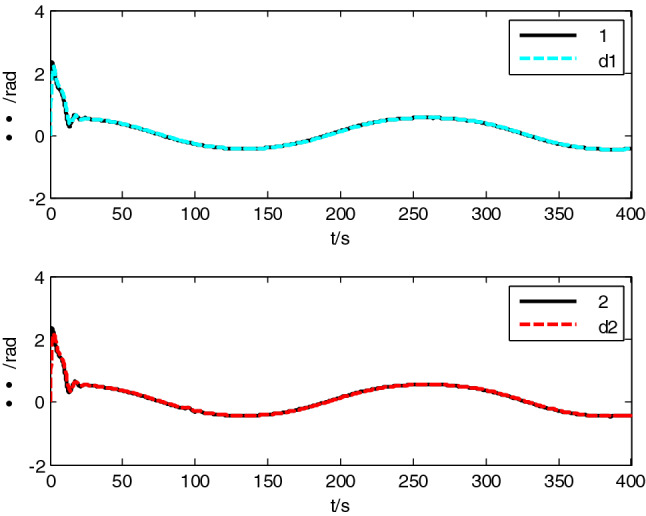
Heading tracking curve.

**Figure 7:**
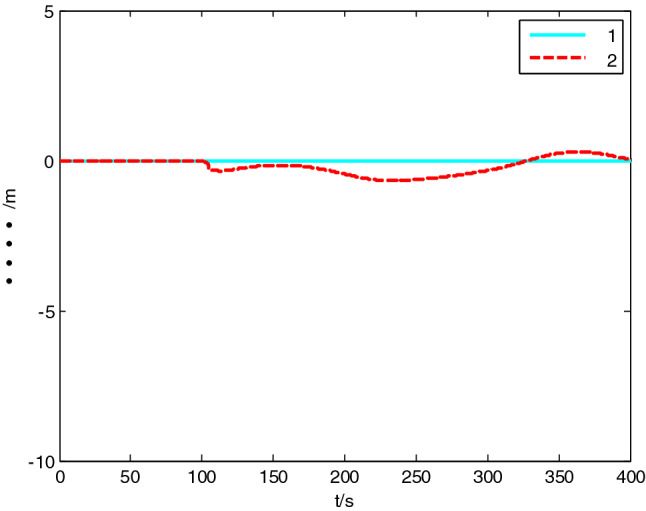
Longitudinal interference.

**Figure 8:**
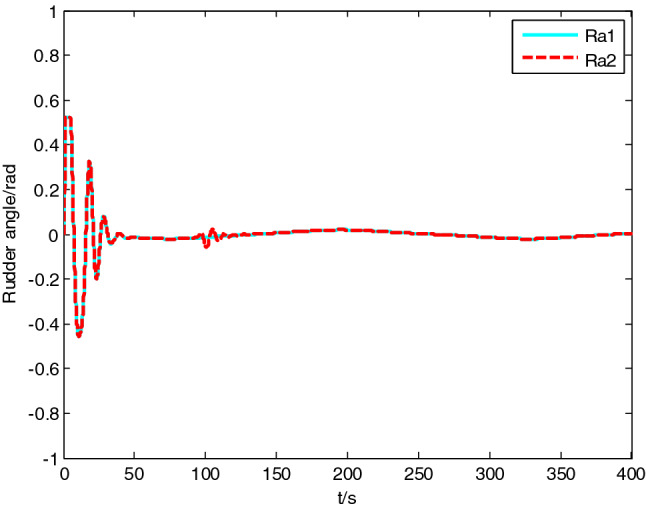
Rudder angle change curve.

**Figure 9:**
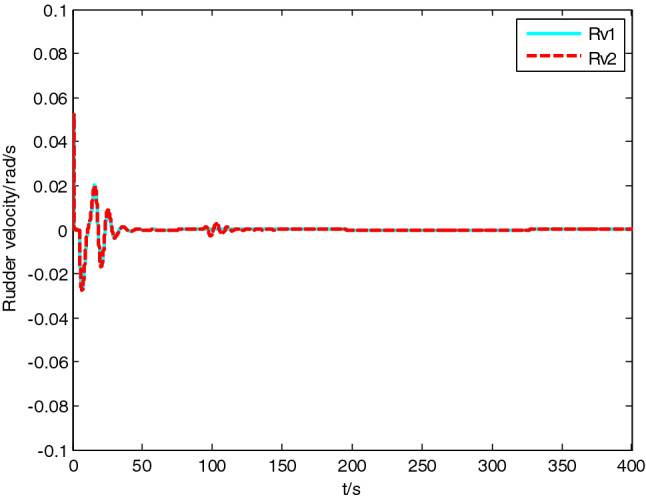
Rudder velocity change curve.

USV had original waypoint (50 m, − 50 m), expected path $x = 100 * \sin (0.005\;{\text{y}})$ , velocity $5\;{\text{m}}/{\text{s}}$, and control parameters $\Delta_{\min } = {15}\;{\text{m}}$, $\Delta_{\max } = 20\;{\text{m}}$, $\kappa = 0.01$, $K_{1c} = 5$, $K_{2c} = 1$, $\sigma = 2$. The resultant velocity of steady-state disturbance was set to 0.5 m/s, and the direction was − *π*/4 rad. Heading control parameters were the same as above. The results of path following are as shown in Figs. 4, 5, 6, 7, 8 and 9. The path following results without disturbance observer and with disturbance observer in this paper are expressed as algorithm 1 and algorithm 2 respectively.

The simulation result shows that, under the effect of the control system designed in this paper, USV could overcome the influence of ocean current, realize the tracking of expected path successfully, and guarantee faster convergence and smaller tracking error. After combining Figs. 4, 5 and 6, it is known that USV is far away from the expected path at the initial stage, and guidance law gives the larger variation of heading. Thereafter, a higher rudder velocity (as shown in Fig. 9) is correspondingly given by the USV heading controller to control the increase of rudder angle (as shown in Fig. 8) and realize the rapid turning. At the stage of stable tracking, there is smaller tracking error and lower rudder velocity, so that rudder angle varies slowly to guarantee high stability and tracking accuracy. It also can be seen from Figs. 4 to Fig. 7, the disturbance observer in this paper can effectively estimate and compensate interference factors, and improve tracking accuracy.

## Vessel test and result analysis

**Figure 10:**
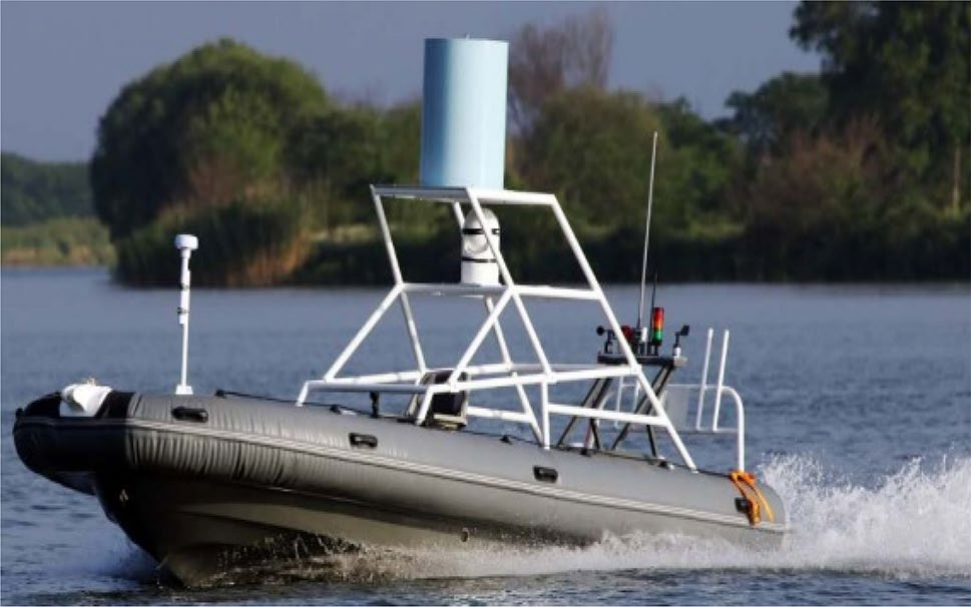
‘Sturgeon’ USV.

**Figure 11:**
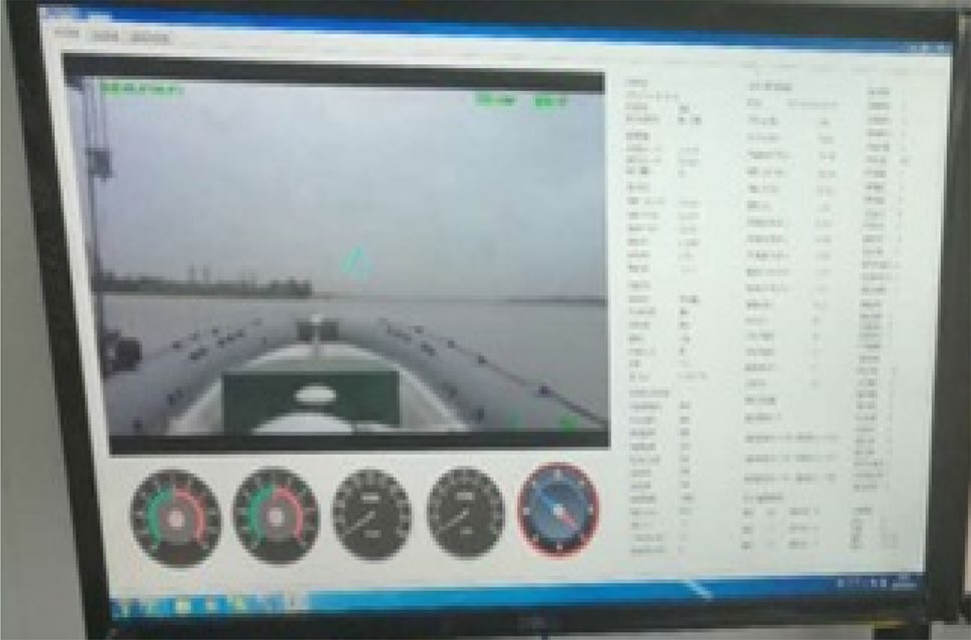
Interface of shore based remote control and monitoring system.

‘Sturgeon’ USV (as shown in Fig. 10) was 7.0 m long and 2.0 m wide, and had the velocity of up to 30 knots and the endurance of 15 h. Based on the idea of modular design, it was equipped with self-developed ship-mounted central control system and ground telemetering control system, and provided with attitude/bearing integrated navigation, wireless communication equipment, photoelectric, radar and other sensor systems, and weapon system, etc. Moreover, it could satisfy the needs of various missions by bearing different loads (Fig. 11).

**Figure 12:**
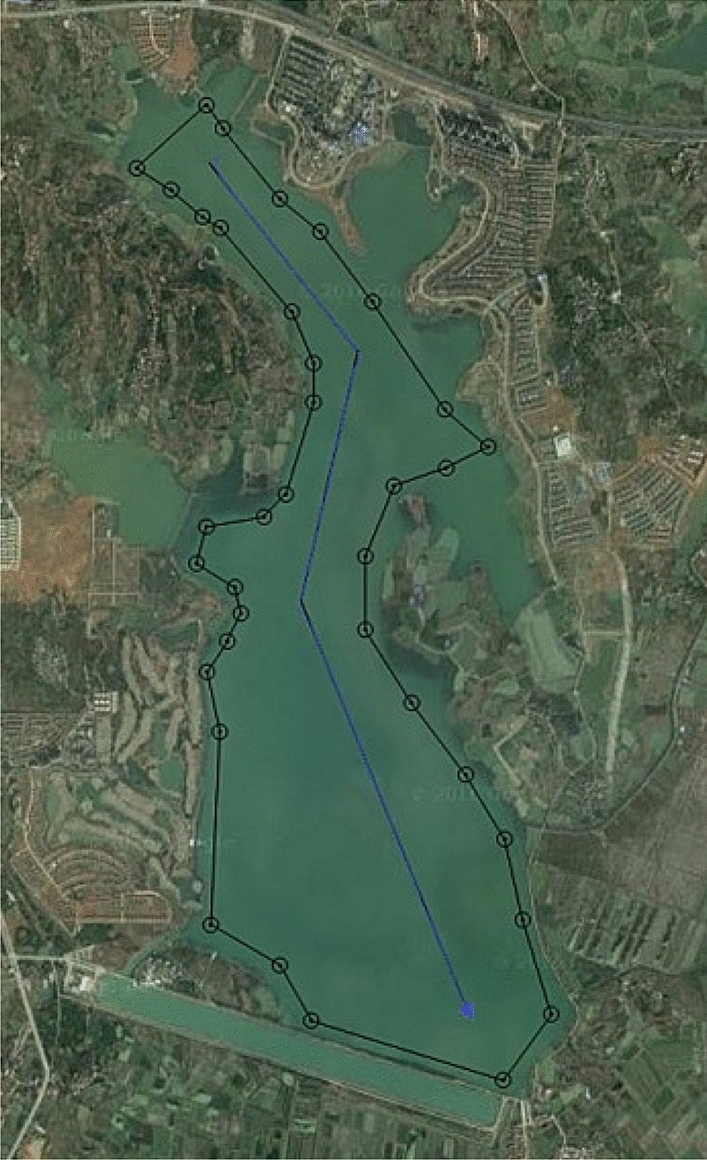
Sturgeon’ USV path following test result.

**Figure 13:**
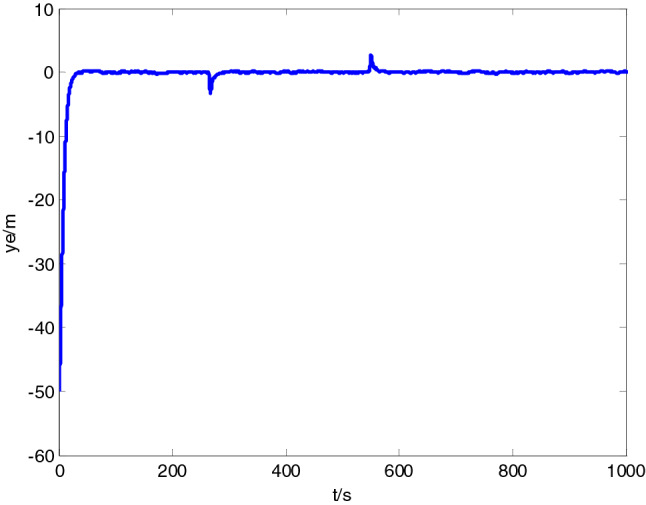
Longitudinal tracking error.

**Figure 14:**
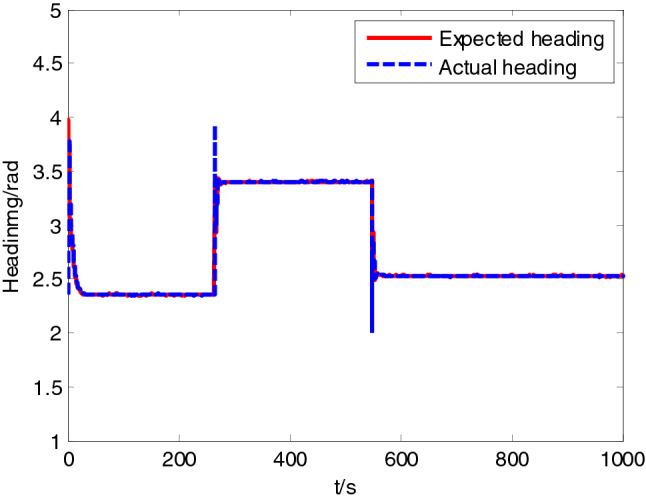
Heading tracking curve.

**Figure 15:**
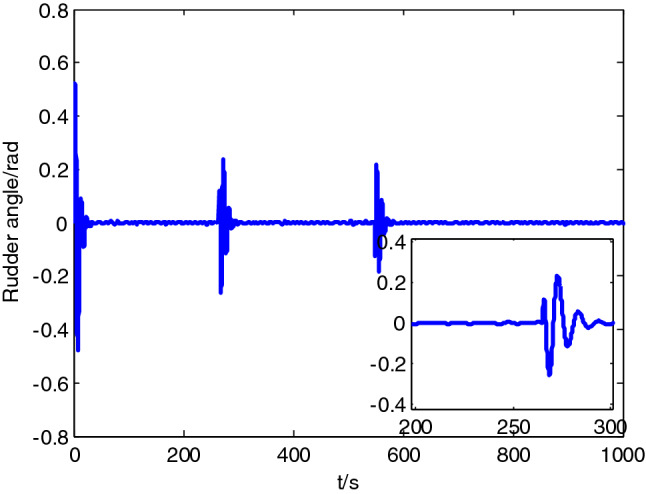
Rudder angle change curve.

Test site was located in Honglian lake, Ezhou. In the test area, an expected path was set to 5 km, while the expected velocity was set to 10 knots. In the process of tracking, ship-mounted central control system could record the information on the state of USV including position, heading, velocity and attitude in a real-time manner. Moreover, ship-mounted wireless communication system could send back the record to the shore-based control station in a real-time manner. USV path following result is shown in Fig. 12, while the state data recorded presented in Figs. 13, 14 and 15.

As shown in test results, ‘Sturgeon’ USV departed from the original position deviated from the expected path (around 50 m), turned at a large rudder angle and rudder velocity, entered the expected path rapidly, and then cruised with smaller tracking error while maintaining the stable heading along the expected path. The vessel test has sufficiently verified the feasibility of this control algorithm in practical engineering applications, and the actual effect of tracking is good.

## Conclusion

In this paper, we designed a complete and superior path following system. According to the cascade system theory, we designed the guidance subsystem and the heading control subsystem respectively, and proved the uniform global asymptotic stability based on the Lyapunov theory.

In terms of guidance law design, while proposing the integral LOS guidance law, we designed a disturbance observer by introducing longitudinal error as the state. Compared with most algorithms based on output feedback compensation, it is more flexible, controllable, and further enhances the accuracy of path following.

After considering the rudder angle constraint and the delay of rudder effect, we designed the control law on the basis of USV motion response model and MPC. Based on simulation and comparison experiments, we found it has obvious advantages compared with the PID controller and the FL controller in ensuring system convergence speed and stability.

In addition, based on the design of the system, we conducted a simulation experiment and further applied the control system to the ‘Sturgeon’ USV for the lake path following test, tracking errors could be rapidly reduced under the action of a stable control law and the USV accurately tracked the expected path. The result is satisfying and also verifies the practical feasibility of the algorithm in engineering applications.

In the following research, the adaptive regulation of the heading control law parameters can be further studied to optimize its control performance and achieve better effect of path following.
